# Cascaded classifiers for confidence-based chemical named entity recognition

**DOI:** 10.1186/1471-2105-9-S11-S4

**Published:** 2008-11-19

**Authors:** Peter Corbett, Ann Copestake

**Affiliations:** 1 Unilever Centre For Molecular Science Informatics, Chemical Laboratory, University Of Cambridge, CB2 1EW, UK; 2 Computer Laboratory, University Of Cambridge, CB3 0FD, UK

## Abstract

**Background:**

Chemical named entities represent an important facet of biomedical text.

**Results:**

We have developed a system to use character-based n-grams, Maximum Entropy Markov Models and rescoring to recognise chemical names and other such entities, and to make confidence estimates for the extracted entities. An adjustable threshold allows the system to be tuned to high precision or high recall. At a threshold set for balanced precision and recall, we were able to extract named entities at an *F *score of 80.7% from chemistry papers and 83.2% from PubMed abstracts. Furthermore, we were able to achieve 57.6% and 60.3% recall at 95% precision, and 58.9% and 49.1% precision at 90% recall.

**Conclusion:**

These results show that chemical named entities can be extracted with good performance, and that the properties of the extraction can be tuned to suit the demands of the task.

## Background

Systems for the recognition of biomedical named entities have traditionally worked on a 'first-best' approach, where all of the entities recognised have equal status, and precision and recall are given roughly equal importance. This does not reflect that fact that precision is of greater importance for some applications, and recall is the key for others. Furthermore, knowing the confidence (in this paper, we use "confidence" to refer to a system's estimate of the probability that a potential named entity is a correct named entity) with which the system has assigned the named entities is likely to be useful in a range of different applications.

Named entities of relevance to biomedical science include not only genes and proteins but also other chemical substances which can be of interest as drugs, metabolites, nutrients, enzyme cofactors, experimental reagents and in many other roles (see [1] for a discussion of chemical terminology). We have recently investigated the issue of chemical named entities [2], by compiling a set of manual annotation guidelines, demonstrating 93% interannotator agreement and manually annotating a set of 42 chemistry papers. In this paper we demonstrate a named entity recogniser that assigns a confidence score to each named entity, allowing it to be tuned for high precision or recall.

Our review of the methods of chemical named entity recognition showed a consistent theme: the use of character-based n-grams to identify chemical names via their constituent substrings [3-5]. This can be a powerful technique, due to systematic and semisystematic chemical names and additional conventions in drug names. However this technique does not cover all aspects of chemical nomenclature.

Much current named entity work uses approaches which combine the structured prediction abilities of HMMs and their derivatives with techniques which enable the use of large, diverse feature sets such as maximum entropy (also known as logistic regression). Maximum Entropy Markov Models, (MEMMs) [6] provide a relatively simple framework for this. MEMMs do have a theoretical weakness, namely the "label bias" problem [7], which has been addressed with the development of Conditional Random Fields (CRFs). CRFs are now a mainstay of the field, being used in a high proportion of entries in the latest BioCreative evaluation [8]. However, despite the label bias problem, MEMMs still attract interest due to practical advantages such as shorter training cycles.

The standard strategy for encoding named-entity recognition in HMMs and similar systems is via BIO coding (or some variant of BIO coding). In this scheme, the text is tokenised, and each token is encoded as O ("outside" – not a part of a named entity), B ("begin") or I ("inside"). Where more than one named entity type exists, the B and I codes can be extended with an entity type. For example, in "dissolved in ethyl acetate." the named entity information can be represented thus: dissolved_O in_O ethyl_B-CM acetate_I-CM ._O.

The framework of HMMs and their successors offers three modes of operation; first-best, n-best and confidence-based. In first-best NER, the Viterbi algorithm is used to identify a single sequence of labels for the target sentence. In n-best operation, the *n *best sequences for the sentence are identified, along with their probabilities, for example by coupling the Viterbi algorithm with A* search. In confidence-based operation, potential entities (with a probability above a threshold) are identified directly, without directly seeking a single optimal labelling for the entire sentence. This is done by examining the probability of the label transitions within the entity, and the forward and backward probabilities at the start and end of the entity. This mode has been termed the Constrained Forward-Backward algorithm [9]. Where a single unambiguous non-overlapping labelling is required, it can be obtained, for example, by identifying cases where the entities overlap, and discarding those with lower probabilities. Note that the set of entities selected in this manner is not guaranteed to be optimal; however, more advanced decoding procedures exist that do guarantee an optimal selection.

Confidence-based extraction has two main advantages. First, it enables the balance between precision and recall to be controlled by varying the probability threshold. Second, confidence-based NER avoids over-commitment in systems where it is used as a preprocessor, since multiple overlapping options can be used as input to later components.

The optimum balance between recall and precision depends on the application of the NER and on the other components in the system. High precision is useful in search even when recall is low when there is a large degree of redundancy in the information in the original documents. High precision NER may also be useful in contexts such as the extraction of seed terms for clustering algorithms. Balanced precision/recall is often appropriate for search, although in principle it is desirable to be able to shift the balance if there are too many/too few results. Balanced precision/recall is also generally assumed for use in strictly pipelined systems, when a single set of consistent NER results is to be passed on to subsequent processing. Contexts where high recall is appropriate include those where a search is being carried out where there is little redundancy (cf [10]) or where the NER system is being used with other components which can filter the results.

One use of our NER system is within a language processing architecture [11] that systematically allows for ambiguity by treating the input/output of each component as a lattice (represented in terms of standoff annotation on an original XML document). This system exploits relatively deep parsing, which is not fully robust to NER errors but which can exploit complex syntactic information to select between candidate NER results. NER preprocessing is especially important in the context of chemistry terms which utilise punctuation characters (e.g., '2,4-dinitrotoluene', '2,4- and 2,6-dinitrotoluene') since failure to identify these will lead to tokenisation errors in the parser. Such errors frequently cause complete parse failure, or highly inaccurate analyses. In our approach, the NER results contribute edges to a lattice which can (optionally) be treated as tokens by the parser. The NER results may compete with analyses provided by the main parser lexicon. In this context, some NER errors are unimportant: e.g., the parser is not sensitive to all the distinctions between types of named entity. In other cases, the parser will filter the NER results. Hence it makes sense to emphasise recall over precision. We also hypothesise that we will be able to incorporate the NER confidence scores as features in the parse ranking model.

An example of the improvements that are available by passing multiple hypotheses from component to component is given by Roth and Yi [12,13]. In their system, results from named entity classification are used in relation classification. By passing multiple hypotheses between the systems, using Integer Linear Programming to perform global inference, they were able to achieve an improvement in performance for both tasks over a more traditional pipelined system. Similarly, Finkel et al. [14] were able to improve the results of Semantic Role Labelling and Recognising Textual Entailment systems by using multiple hypotheses from their underlying named entity recognition and PCFG parsing components.

One motivation for our focus on high-recall capable NER is its intended use in the Royal Society of Chemistry's editing workflows for their Project Prospect system [15], in which chemical named entity recognition is used to produce semantically-enriched journal articles. In this situation, high recall is desirable, as false positives can be removed in two ways; by removing entities where a chemical structure cannot be assigned, and by having them checked by a technical editor. False negatives are harder to correct. A study by Alex et al. [16] on the use of NLP components in the curation of biological databases showed a curator preference for high-recall over high-F score NER. They speculate that different curators may have different preferences for precision and recall, and suggest that it would be useful to develop a system which would allow curators to filter information dynamically based on confidence values.

The use of confidence-based recognition has been demonstrated with CRFs in the domain of contact details [9], and using HMMs in the domain of gene annotation [10]. In the latter case, the LingPipe toolkit was used in the BioCreative 2 evaluation without significant adaptation. Although only 54% precision was achieved at 60% recall (the best systems were achieving precision and recall scores in the high eighties), the system was capable of 99.99% recall with 7% precision, and 95% recall with 18% precision, indicating that very high recall could be obtained in this difficult domain.

Another potential use of confidence-based NER is the potential to rescore named entities. In this approach, the NER system is run, generating a set of named entities. Information obtained about these entities throughout the document (or corpus) that they occur in can then be used in further classifiers. We are not aware of examples of rescoring being applied to confidence-based NER, but there are precedents using other modes of operations. For example, Krishnan and Manning [17] describe a system where a first-best CRF is used to analyse a corpus, the results of which are then used to generate additional features to use in a second first-best CRF. Similarly, Yoshida and Tsujii [18] use an n-best MEMM to generate multiple analyses for a sentence, and re-rank the analyses based on information extracted from neighbouring sentences. Ji and Grishman [19-21] use feedback from coreference resolution, relation extraction and event extraction systems to rescore the output of an n-best HMM Chinese name tagger.

Therefore, to explore the potential of these techniques, we have produced a chemical NER system that uses a MEMM for confidence-based extraction of named entities, with an emphasis on the use of character-level n-grams, and a rescoring system.

## Methods

### Corpus

Previously, we have produced a set of annotation guidelines for chemical named entities, and used them to annotate a set of 42 chemistry papers [2]. In that study, inter-annotator agreement was tested on 14 of these, and found to be 93%. The annotation guidelines specified five classes of named entity, which are detailed in Table 1. The annotation was performed on untokenised text. When tokenised with our tokeniser, the corpus was found to contain 129576 tokens (including punctuation) in total.

**Table 1:** Named Entity Types

Type	Description	Example	*n*_*Ch*_	*n*_*PM*_
CM	compound	citric acid	6865	4494
RN	reaction	methylation	288	401
CJ	adjective	pyrazolic	60	87
ASE	enzyme	demethylase	31	181
CPR	prefix	1,3-	53	21

*n*_*Ch *_= number in Chemistry corpus, *n*_*PM *_= number in PubMed corpus.

To test the applicability of the method to a different corpus, we retrieved 500 PubMed abstracts and titles, and annotated them using the same methods. The abstracts were acquired using the query metabolism [Mesh] AND drug AND hasabstract. This produced a diverse set of abstracts spanning a wide range of subject areas, but which contain a higher proportion of relevant terms than PubMed overall. 445 out of 500 abstracts contained at least one chemical named entity, whereas 249 contained at least ten. Notably, the ASE class was more common in the PubMed corpus than in the chemistry papers, reflecting the importance of enzymes to biological and medical topics. This corpus was found to contain 135197 tokens (including punctuation) in total.

In the current study, we have left out the named entity type CPR, as it is rare (<1%) and causes difficulties with tokenisation. This entity type covers cases such as the "1,3-" in "1,3-disubstituted", and as such requires the "1,3-" to be a separate token or token sequence. However, we have found that recognition of the other four classes is improved if words such as "1,3-disubstituted" are kept together as single tokens. Therefore it makes sense to treat the recognition of CPR as an essentially separate problem – a problem that will not be addressed here.

### External Resources

Chemical names were extracted from the chemical ontology ChEBI [22], and a standard English word list was taken from/usr/share/dict/wordson a Linux system. This dictionary was chosen as it contains inflectional forms of English words. Our system does not perform stemming, partly because suffixes are often good cues as to whether a word is chemical or not. A list of chemical element names and symbols was also compiled. To overcome the shortage of entities of type ASE, a list of words from enzyme names ending in '-ase' was extracted from the Gene Ontology [23], and manually sorted into words of type ASE, and words not of type ASE.

### Overview of operation

The text is tokenised before processing; this is done using the tokeniser described in our previous work [2], which is adapted to chemical text.

Our system uses three groups of classifiers to recognise chemical names. The first classifier – the 'preclassifier' – uses character-level n-grams to estimate the probabilities of whether tokens are chemical or not. The output of this classification is combined with information from the suffix of the word, and is used to provide features for the MEMM.

The second group of classifiers constitute the MEMM proper. Named entities are represented using a BIO-encoding, and methods analogous to other confidence-based taggers [9,10] are used to estimate the conditional probability of tag sequences corresponding to named entities. The result of this is a list of potential named entities, with start positions, end positions, types and probabilities, where all of the probabilities are above a threshold value. A small set of hand-written filtering rules is used to remove obvious absurdities, such as named entities ending in the word "the", and simple violations of the annotation guidelines, such as named entities of type ASE that contain whitespace. These filtering rules make very little difference at recall values up to about 80% – however, we have found that they are useful for improving precision at very high recall.

The third group of classifiers – one per entity type – implement a rescoring system. After all of the potential entities from a document have been generated, a set of features is generated for each entity. These features are derived from the probabilities of other entities that share the same text string as the entity, from probabilities of potential synonyms found via acronym matching and other processes, and most importantly, from the pre-rescoring probability of the entities themselves.

### Overview of training

A form of training conceptually similar to cross-validation is used to train the three layers of classifiers. To train the overall system, the set of documents used for training is split into three parts. Two thirds are used to train a MEMM, which is then used to generate training data for the rescorer using the held-out last third. This process is repeated another two times, holding out a different third of the training data each time. Finally, the rescorer is trained using all of the training data generated by this procedure, and the final version of the MEMM is generated using all of the training data. This procedure ensures that both the MEMM and the rescorer are able to make use of all of the training data, and also that the rescorer is trained to work with the output of a MEMM that has not been trained on the documents that it is to rescore.

A similar procedure is used when training the MEMM itself. The available set of documents to use as training data is divided into half. One half is used to train the preclassifier and build its associated dictionaries, which are then used to generate features for the MEMM on the other half of the data. The roles of each half are then reversed, and the same process is applied. Finally, the MEMM is trained using all of the generated features, and a new preclassifier is trained using all of the available training data. It should be noted that the dictionaries extracted during the training of the preclassifier are also used directly in the MEMM.

### The character n-gram based preclassifier

During the training of the preclassifier, sets of tokens are extracted from the hand-annotated training data. A heuristic is used to classify these into 'word tokens' – those that contain two consecutive lowercase letters, and 'nonword tokens' – those that do not (this class includes many acronyms and chemical formulae). The n-gram analysis is only performed upon 'word tokens'. This is a simple heuristic that excludes many irrelevant tokens from consideration, but it does not aim to be comprehensive.

The token sets that are compiled are chemical word tokens (those that only appear inside named entities), nonchemical word tokens (those that do not appear in entities), chemical nonword tokens, nonchemical nonword tokens and ambiguous tokens – those that occur both inside and outside of named entities. A few other minor sets are collected to deal with tokens related to such proper noun-containing entities as 'Diels-Alder reaction'.

Some of this data is combined with external dictionaries to train the preclassifier, which uses Markov models of chemical names and nonchemical words, using 4-grams of characters and modified Kneser-Ney smoothing, as described in [5]. In our experience with various n-gram techniques, using 5-grams and higher offers only a slight advantage at best and is counter-productive at worst. The set of 'chemical word tokens' is used as a set of positive examples, along with tokens extracted from ChEBI, a list of element names and symbols, and the ASE tokens extracted from the GO. The negative examples used are the extracted 'nonchemical word tokens', the non-ASE tokens from the GO and tokens taken from the English dictionary – except for those that were listed as positive examples. This gets around the problem that the English dictionary contains the names of all of the elements and a number of simple compounds such as 'ethanol'.

During operation, n-gram analysis is used to calculate a score for each word token, of the form:

ln(*P*(*token*|*chem*)) - ln(*P*(*token*|*nonchem*))

If this score is above zero, the preclassifier classifies the token as chemical and gives it a tentative type, based on its suffix. This can be considered to be a "first draft" of its named entity type. For example tokens ending in "-ation" are given the type RN, whereas those ending in "-ene" are given type CM.

### The MEMM

The MEMM is a first-order MEMM, in that it has a separate maximum-entropy model for each possible preceeding tag. No information about the tag sequence was included directly in the feature set. We use the OpenNLP MaxEnt toolkit [24] for maximum-entropy classification.

The feature set for the MEMM is divided into three types of features; type 1 (which apply to the token itself), type 2 (which can apply to the token itself, the previous token and the next token) and type 3 (which can act as type 2 features, and which can also form bigrams with other type 3 features). An example type 1 feature would be 4G=ceti, indicating that the 4-gram ceti had been found in the token. An example type 2 feature would be c-1:w=in, indicating that the previous token was 'in'. An example bigram constructed from type 3 features would be bg:0:1:ct=CJ_w=acid, indicating that the preclassifier had classified the token as being of type CJ, and having a score above zero, and that the next token was 'acid'.

Type 1 features include 1, 2, 3 and 4-grams of characters found within the token, whether the token appeared in any of the word lists, and features to represent the probability and type given by the preclassifier for that token. Type 2 features include the token itself with any terminal letter 's' removed, the token converted to lowercase (if it matched the regex .*[a-z][a-z].*), and a three-character suffix taken from the token. The token itself was usually used as a type 2 feature, unless it was short (less than four characters), or had been found to be an ambiguous token during preclassifier training, in which case it was type 3 (i.e. licensed to occur as a bigram with other type 3 features). Other type 3 features include a word shape feature, and tentative type of the token if the preclassifier had classed it as chemical.

A few other features were used to cover a few special cases, and were found to yield a slight improvement during development.

After generating the features, the least informative features are removed by discarding all of those with a *G *less than or equal to 0.25, where



This was found during development to have only a very small beneficial effect on the performance of the classifier, but it did make training faster and produced smaller models. This largely removed rare features which were only found on a few non-chemical tokens.

### The rescorer

The rescoring system works by constructing four maximum entropy classifiers, one for each entity type. The output of these classifiers is a probability of whether or not a potential named entity really is a correct named entity of the respective class. The generation of features is done on a per-document basis.

The key features in the rescorer represent the probability of the potential entity as estimated by the MEMM. The raw probability *p *is converted to the logit score

*l *= ln(*p*) - ln(1 - *p*)

This mirrors the way probabilities are represented within maximum entropy (*aka *logistic regression) classifiers. If *l *is positive, *int*(*min*(15.0, *l*) * 50) instances of the feature conf+ are generated, and a corresponding technique is used if *l *is negative. We found that 15.0 was a good threshold by experimentation on development data: papers annotated during trial runs of the annotation process. Before generating further features, it is necessary to find entities that are 'blocked' – entities that overlap with other entities of higher confidence. For example, consider "ethyl acetate", which might give rise to the named entity "ethyl acetate" with 98% confidence, and also "ethyl" with 1% confidence and "acetate" with 1% confidence. In this case, "ethyl" and "acetate" would be blocked by "ethyl acetate".

Further features are generated by collecting together all of the unblocked potential entities of a type that share the same string, calculating the maximum and average probability, and calculating the difference between the *p *and those quantities. It is important to consider only unblocked entities here, as doing this without regards for blocking causes problems. In a document containing both "ethyl acetate" and "ethyl group", it would be detrimental to allow the low confidence for the "ethyl" in "ethyl acetate" to lower the confidence of the "ethyl" in "ethyl group".

Some acronym and abbreviation handling is also performed. The system looks for named entities that are surrounded by brackets. For each of these, a list of features is generated that is then given to every other entity of the same string. If there is a potential entity to the left of the bracketed potential abbreviation, then features are generated to represent the probability of that potential entity, and how well the string form of that entity matches the potential abbreviation. If no potential entity is found to match with, then features are generated to represent how well the potential abbreviation matches the tokens to the left of it. By this method, the rescorer can gather information about whether a potential abbreviation stands for a named entity, something other than a named entity – or whether it is not an abbreviation at all, and use that information to help score all occurrences of that abbreviation in the document.

## Results and discussion

The systems were evaluated by 3-fold cross-validation methodology, whereby the data was split into three equal folds (in the case of the chemistry papers, each fold consists of one paper per journal. For the PubMed abstracts, each fold consists of one third of the total abstracts). For each fold, the system was trained on the other two folds and then used to generate a list of putative named entities on that fold. The putative named entities generated in this fashion were then pooled into a single list.

In this list, the start position, end position, type and confidence score of each putative named entity was recorded. This list was sorted in order of confidence – most confident first – and each entity was classified as a true positive or a false positive according to whether an exact match (start position, end position and type all matched perfectly) could be found in the annotated corpus. Also, the number of entities in the annotated corpus was recorded.

**Figure 1:**
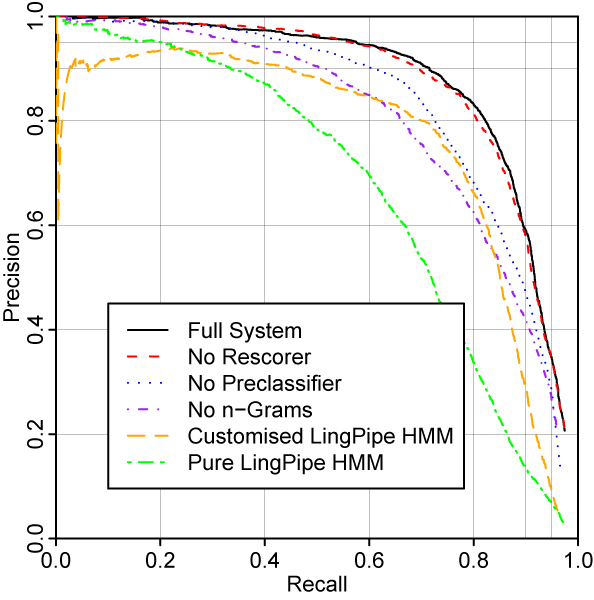
Evaluation on chemistry papers.

Precision/recall curves were plotted from these lists by selecting the first *n *elements, and calculating precision and recall taking all of the elements in this sublist as true or false positives, and all the entities in the corpus that were not in the sublist as false negatives. The value of *n *was gradually increased, recording the scores at each point. The Mean Average Precision (MAP) was calcuated as the average of precisions computed after each true positive in turn (named entities that were not detected at all were treated as having a precision of zero). This value is approximately equal to the area under the precision/recall curve. We also consider some single-point values: the precision at a threshold that sets the recall to 90%, the recall at a threshold that sets the precision to 95%, and the precision, recall and *F *at a threshold of 0.3. The results of this evaluation on the corpus of chemistry papers are shown in Figure 1 and Table 2. The full system achieves 57.6% recall at 95% precision and 58.9% precision at 90% recall. At a confidence threshold of 0.3, the system achieves 78.7% precision and 82.9% recall (*F *= 80.7%). Also shown are the results of successively eliminating parts of the system. "No Rescorer" removes the rescorer. In "No Preclassifier", the preclassifier is disabled (we disable the preclassifier rather than the MEMM here, as a preclassifier-only system would only be able to recognise single-token named entities that contain consecutive lowercase characters, which constitute only 55% of the named entities in the corpus. The MEMM, by contrast, is not fundamentally limited in this manner), and all of the dictionaries extracted during the training of the preclassifier are also disabled. Finally, in "No n-Grams", the 1-, 2-, 3- and 4-grams used directly by the MEMM are also disabled, showing the results of using a system where no character-level n-grams are used at all. These modifications apply successively – for example, in the "No n-Grams" case the rescorer and preclassifier are also disabled. These results validate the the cascade of classifiers, and underline the importance of character-level n-grams in chemical NER.

**Table 2:** *F *scores (at confidence threshold of 0.3) and Mean Average Precision (MAP) values for Figs. 1–5.

Corpus	System	MAP	*F*
Chemistry	Full	87.1%	80.8%
Chemistry	No Rescorer	86.8%	81.0%
Chemistry	No Preclassifier	82.7%	74.8%
Chemistry	No n-Grams	79.2%	72.2%
Chemistry	Custom LingPipe	75.9%	74.6%
Chemistry	Pure LingPipe	66.9%	63.2%
Chemistry	No Overlaps	82.9%	80.8%
Chemistry	CM	87.0%	81.2%
Chemistry	RN	74.5%	73.4%
Chemistry	CJ	90.0%	92.0%
Chemistry	ASE	17.4%	36.2%
PubMed	Full	86.1%	83.2%
PubMed	No Rescorer	83.3%	79.1%
PubMed	No Preclassifier	81.4%	73.4%
PubMed	No n-Grams	77.6%	70.6%
PubMed	Custom LingPipe	78.6%	75.6%
PubMed	Pure LingPipe	71.9%	66.1%
PubMed	CM	85.6%	82.3%
PubMed	RN	95.3%	93.2%
PubMed	CJ	78.7%	83.1%
PubMed	ASE	83.4%	86.0%

We also show comparisons to an HMM-based approach, based on LingPipe 3.4.0 [25]. This is essentially the same system as we described in [2], but operating in a confidence-based mode. The HMMs used make use of character-level n-grams, but do not allow the use of the rich feature set used by the MEMM. The line "Customised LingPipe HMM" shows the system using the custom tokenisation and ChEBI-derived dictionary used in the MEMM system, whereas the "Pure LingPipe HMM" shows the system used with the default tokeniser and no external dictionaries. In the region where precision is roughly equal to recall (mimicking the operation of a first-best system), the fact that the MEMM-based system outperforms an HMM is no surprise. However, it is gratifying that a clear advantage can be seen throughout the whole recall range studied (0–97%), indicating that the training processes for the MEMM are not excessively attuned to the first-best decision boundary. This increased accuracy comes at a price in the speed of development, training and execution.

It is notable that we were not able to achieve extremes of recall at tolerable levels of precision using any of the systems, whereas it was possible for LingPipe to achieve 99.99% recall at 7% precision in the BioCreative 2006 evaluation. There are a number of reasons for this. The first is that the tokeniser used in all systems apart from the "Pure LingPipe HMM" system tries in general to make as few token boundaries as possible; this leads to some cases where the boundaries of the entities to be recognised in the test paper occur in the middle of tokens, thus making those entities unrecognisable whatever the threshold. Other factors that may have had an influence include the quantity of training data, differences between chemical and gene names, and the fact that the sentences used in BioCreative were selected using a classifier to control the rate at which gene names occurred in that corpus. [8]

**Figure 2:**
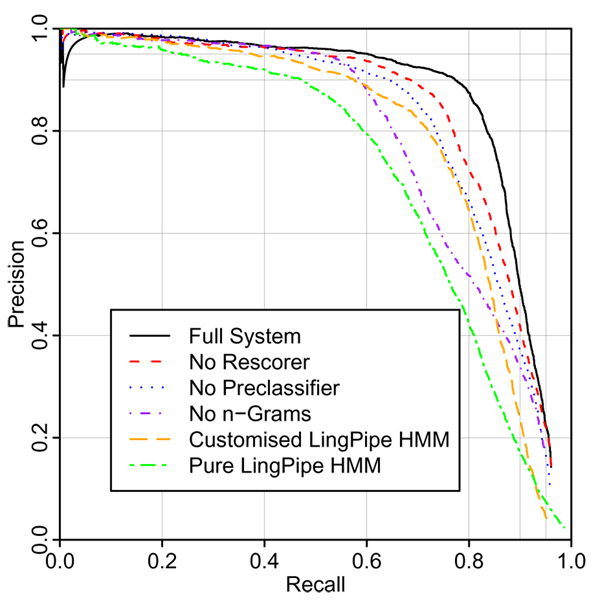
Evaluation on PubMed abstracts.

Figure 2 shows the results of running the system on the set of annotated PubMed abstracts described earlier. The full system achieves 60.3% recall at 95% precision, 49.1% precision at 90% recall. At a confidence threshold of 0.3, the system achieves 85.0% precision and 81.6% recall (*F *= 83.2%). In PubMed abstracts, it is common to define ad-hoc abbreviations for chemicals within an abstract (e.g., the abstract might say 'dexamethasone (DEX)', and then use 'DEX' and not 'dexamethasone' throughout the rest of the abstract). The rescorer provides a good place to resolve these abbreviations, and thus has a much larger effect than in the case of chemistry papers where these ad hoc abbreviations are less common. Chemistry papers conventionally number compounds and indicate coreference between compounds with numbers rather than abbreviations. Since we can retrieve these almost perfectly by straightforward manipulation of the document structure, we do not include such references in our annotations and results. However it is worth noting that the effective performance of NER on chemistry texts is considerably higher when these coreferences are taken into account. It is also notable that the maximum recall is lower for the PubMed abstracts. One system – the "Pure LingPipe HMM", which uses a different, more aggressive tokeniser from the other systems – has a clear advantage in terms of maximum recall, showing that overcautious tokenisation limits the recall of the other systems.

In some cases the system gives "spikes" of lowered precision at very low recall, indicating that it can occasionally be overconfident, and assign very high confidence scores to incorrect named entities. Neither corpus contains enough data for the results to reach a plateau – using additional training data is likely to give improvements in performance.

**Figure 3:**
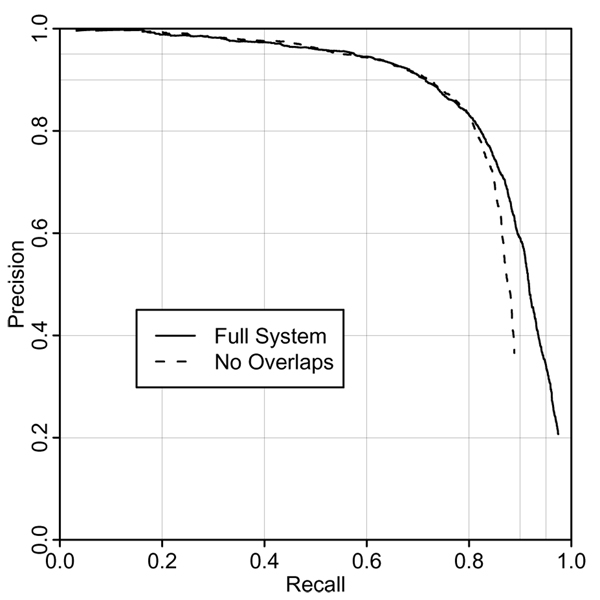
Evaluation on chemistry papers, showing effects of disallowing overlapping entities.

The "No Overlaps" line in Figure 3 shows the effect of removing "blocked" named entities (as defined in section 3.6) prior to rescoring. This simulates a situation where an unambiguous inline annotation is required – for example a situation where a paper is displayed with the named entities being highlighted. This condition makes little difference at low to medium recall, but it sets an effective maximum recall of 90%. The remaining 10% of cases presumably consist of situations where the recogniser is finding an entity in the right part of the text, but making boundary or type errors.

**Figure 4:**
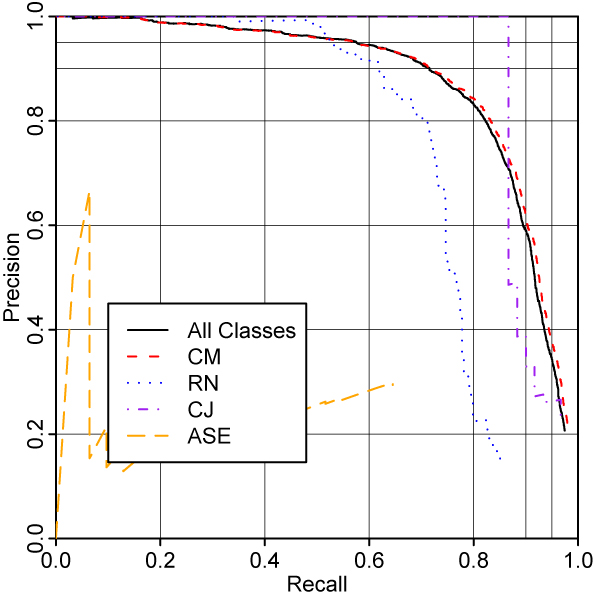
Evaluation on chemistry papers, showing performance on different named entity classes.

**Figure 5:**
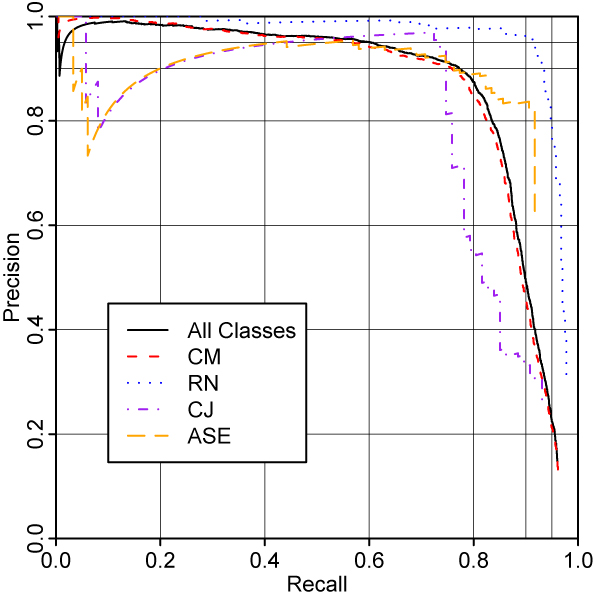
Evaluation on PubMed abstracts, showing performance on different named entity classes.

Figure 4 shows the performance of the full system on chemistry papers, evaluating each named entity class separately. As expected, the performance of the system on CM closely mirrors the overall performance, with RN being in general handled worse and CJ being handled better. The performance on ASE is very poor. Figure 5 shows the same analysis for the PubMed abstracts. Here, the performace on ASE is greatly improved, presumably due to those entities being much more common in this corpus. RN, too, is easier to recognise in this corpus; we suspect that this is due to there being fewer complex reaction names such as "Diels-Alder reaction".

## Conclusion

We have demonstrated that MEMMs can be adapted to recognise chemical named entities, and that the balance between precision and recall can be tuned effectively, at least in the range of 0 – 95% recall. The MEMM system is available as part of the OSCAR3 chemical named entity recognition system [26].

## Competing interests

The authors declare that they have no competing interests.

## Authors' contributions

AC conceived the original idea and supervised all steps of the work. PC wrote the software, annotated the PubMed abstracts, performed the experiments and analysed the results. Both authors wrote the manuscript and approved the final version.
